# Probing Mixed-Genotype Infections I: Extraction and Cloning of Infections from Hosts of the Trypanosomatid *Crithidia bombi*

**DOI:** 10.1371/journal.pone.0049046

**Published:** 2012-11-14

**Authors:** Rahel Salathé, Martina Tognazzo, Regula Schmid-Hempel, Paul Schmid-Hempel

**Affiliations:** Institute of Integrative Biology (IBZ), ETH Zürich, Zürich, Switzerland; University of Leeds, United Kingdom

## Abstract

We here present an efficient, precise and reliable method to isolate and cultivate healthy and viable single *Crithidia bombi* cells from bumblebee faeces using flow cytometry. We report a precision of >99% in obtaining single trypanosomatid cells for further culture and analysis (“cloning”). In the study, we have investigated the use of different liquid media to cultivate *C. bombi* and present an optimal medium for obtaining viable clones from all tested, infected host donors. We show that this method can be applied to genotype a collection of clones from natural infections. Furthermore, we show how to cryo-preserve *C. bombi* cells to be revived to become infective clones after at least 4 years of storage. Considering the high prevalence of infections in natural populations, our method provides a powerful tool in studying the level and diversity of these infections, and thus enriches the current methodology for the studies of complex host-parasite interactions.

## Introduction

In mixed-genotype infections a given host individual is infected by more than one genotype of the same parasite. This can be a result of several processes such as co-infection, mutations, or genetic exchange. There is an increasing awareness that mixed-genotype infections are very common and can be observed in many different systems [1], [2]. These mixed-genotype infections have consequences for both ecology and evolutionary dynamics of host-parasite interactions. For example, co-infecting parasite genotypes may compete for host resources and transmission opportunities, and such competition may furthermore be mediated by the host's immune system. In addition, competition between co-infecting parasite genotypes can have effects on the expressed virulence, which are predicted to either increase [3] or decrease [4], [5] depending on which genotypes infect and what kind of interaction among the parasites and host defences are assumed. For example, experimental co-infections of the rodent malaria, *Plasmodium chabaudi*, in both host and vector, vary in outcome [6], [7], and mixed infections can thereby have lower transmission potential [8], and the success of each genotype depends on whether it infects as a majority or minority strain in the mouse host to begin with [9].

Hence there is little doubt that the analysis of mixed-genotype infections is important for our understanding of evolutionary parasitology [10]. Even though an increasing number of studies address this question [2], [11]–[17], the theoretical treatment of mixed genotype-infections still outstrips the empirical studies. A recurrent problem in field studies or non-experimental assays is to identify the constituent genotypes of a mixed infection. So far, several methods have been used. In *Toxoplasma gondii*, for example, these include growth in cell cultures followed by the typing of SNPs [18], or partial sequencing of polymorphic genes [15]. More sophisticated techniques, involving base-specific cleavage and mass spectrometry (for *Neissera meningitidis*; [19]), or a primer-specific mispair extension protocol (for Hepatitis C-virus; [20]) have also been applied. For most ecological studies, however, such methods are beyond what can be managed. Here, we describe and validate a method that works with high reliability and high resolution and that can manage relevant sample sizes if needed.

*Crithidia bombi* (Trypanosomatidae) [21] is an obligatory gut parasite of bumblebees (*Bombus* spp.). It is an intestinal parasite of bumblebees that infects when cells of the parasite are imbibed from contaminated flowers [22] or when picked up from contaminated surfaces in the nest. This parasite not only serves as a convenient model to study host-parasite interactions [23] but also affects important pollinators of temperate and cool habitats [24]. Among those, *Bombus terrestris* L. is heavily used a commercial pollinator [25], as it can be hosted for its entire life cycle in the laboratory. Bumblebees are primitively social insects, where young queens emerge in spring to found a colony on their own. The colony subsequently grows in worker numbers over the season. After several weeks, the colony produces sexuals (drones and daughter queens) that leave the nest, mate, and only the mated daughter queens then go into hibernation to start the next-generation colonies in the following spring [26]. Infections with *C. bombi* shorten the life span of stressed workers [27] and severely reduce the fitness of founding queens in spring [28]. Previous studies have shown that *C. bombi* is common in natural populations of its hosts [29], [30], that there are genotype-genotype interactions with its host [31], and that multiple infections are found at high prevalence [30], [32], [33]. Furthermore, within-host competition [34] and genetic exchange between co-infecting strains [35] is known to occur.

Methods to clone and cultivate *C. bombi* in culture media had already previously been developed [36]–[39] and used successfully to infect experimental animals with defined clones [34], [35], [40]–[43]. We here show that flow-cytometric-single-cell-sorting, combined with the presented medium, is an even more efficient technique for establishing single-genotype (clonal) cultures of *C. bombi* (used in [34], [35], [42], [43]). We demonstrate that the precision of single-cell sorting is very high and that the method proves to be a valuable tool to establish and study a large number of clones from environmental samples or in vitro experiments in a short time. In addition, we report that *C. bombi* can be stored for extended periods of time and also provide some data on microsatellite evolution during long-term cultivation.

## Materials and Description of Method

### Evaluation of methods

Prior to settling for the method described here, a number of alternatives to isolate and cultivate *C. bombi* were tested. We describe these tests to illustrate the breadth and scope of the study.

### Single cell sorting and cultivation

**Figure 1:**
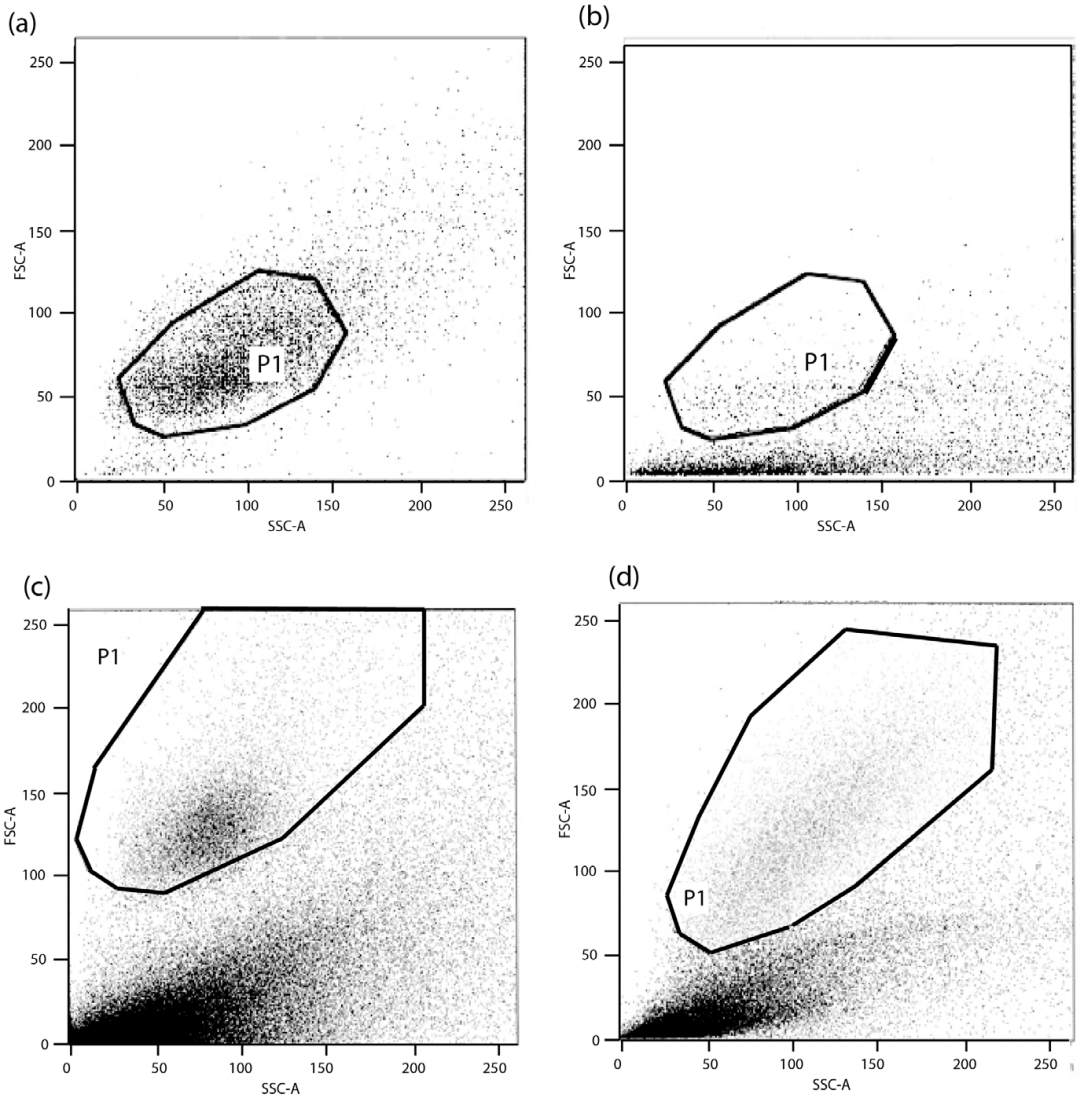
Scatter plot of defined cell populations targeted for sorting. Axes represent relative differences in size of cells (FSC-A), and relative differences in complexity or granularity of cells (SSC-A). The envelopes (solid lines) define a “gate” (P1) from within which cells will be collected by the procedure. The examples in the panels show (a) the gate for the pure clone no. 08.076, which is a cryo-preserved clone from [36], [37]. Gates (b)–(d) refer to *C. bombi* cells from raw faeces. For (a) and (b), the defined collection gates P1 are identical;(c) and (d) have adjusted P1 gates, as different clones can vary in shape and granularity. Only cells within a defined gate will be selected and sorted into the well plates. Gates can be defined and adjusted based on how variable the targeted population is. All additional cell populations contained in the sample (noise spreading along the SSC-A axis) which could be anything from microbial or fungal origin to cell debris, will be excluded. Note that this noise is much less substantial in the pure clone (a), where the only noise could be dead cells, compared to the faeces samples, which contain cells of various origins.

Faeces from 31 infected spring queens of *B. terrestris* from two locations in Switzerland (13 from Neunforn TG, 18 from Aesch BL; locations approx. 100 km apart; no specific permits were required for these samplings, nor is the species used here protected) were collected in early spring 2008 and their faeces checked for live cells of *C. bombi*. 100 µl of full FP culture medium (see Information S1) were added to each sample. Then, faeces were stored at 4°C until further use (but not longer than a few days). Shortly before sorting, samples were centrifuged at 1,000 rpm for 10 min. After discarding the supernatant, samples were kept on ice. Immediately before starting the sorting process, 1 ml of PBS buffer was added to the sample. Each sample was cleaned from larger particles using disposable filters (CellTrics 50 µl; Partec, Görlitz, Germany) and then submitted to a BD FACSAria™ flow cytometer setup for single cell sorting. Single cells were sorted into 96-well plates (Multiple Well Plate 96-Well Flat Bottom with Lid; Sarstedt, Newton, USA), each well containing 100 µl of medium, using forward and side scatter only (indicating relative differences in size of cells and relative differences in internal complexity or granularity of cells, respectively). Gating parameters were determined using a pure clone of *C. bombi* established by traditional cloning [36]. A relatively large gate (designated “P1”) was chosen to allow the collection of cells with a large diversity of sizes and complexity (Fig. 1A). After sorting, plates were kept at 27°C and 3% CO_2_ for 10–14 days. After one week, wells were checked for *C. bombi* using an inverse microscope (Nikon). For further analysis, one randomly selected clone per bee was then transferred into a 25 ml Falcon® tissue culture flask (Becton Dickinson Labware; New Jersey, USA) containing 5 ml of medium. After growing to large cell numbers, these cultures were either used for genetic analysis or long-term storage.

### Long-term storage

After the FACS-sorting, we prepared the cultured parasites for long-term storage as follows: 300 µl of 50% Glycerol was added to 1.5 ml tubes (Sarstedt) and autoclaved. 700 µl of culture was then added to the tubes. Tubes were put into a “Mr. Frosty” (Cryo 1°C Freezing Container; Nalgene Labware, USA) and gradually frozen as suggested by the manufacturer. Samples were then transferred into liquid nitrogen or remained in the freezer at −80°C. To re-culture the clones, the content of the tube was thawed and then centrifuged at 1,000 rpm for 10 min. After discarding the supernatant, the pellet was washed with 500 µl of fresh medium, centrifuged at 1,000 rpm for 10 min, and the supernatant discarded. The pellet was then re-suspended in a small amount of fresh medium and eventually transferred into a culture flask containing 5 ml of medium.

### Genetic analysis

To prepare the cells for DNA extraction, 1 ml of culture was centrifuged at 1,000 rpm for 10 min and the supernatant discarded. The pellet was washed by adding 500 µl of PBS, centrifuging at 1,000 rpm for 10 min with subsequent discarding the supernatant. Genomic DNA was extracted using the Qiagen DNeasy Blood & Tissue Kit following their protocol for cultured cells. Multi-locus genotypes were determined using five microsatellites described by Schmid-Hempel & Reber Funk [32], adopting a slightly different PCR protocol as follows. Using labelled primers, the five loci were amplified in two different multiplex PCR reactions (Msat1 and Msat2) with the following specifications: Msat 1 amplified loci *Cri4*, *Cri2F1*0 and *Cri4G9A* using an annealing temperature of 48°C, and Msat 2 amplified loci *Cri16* and *Cri1B6*, using a annealing temperature of 52°C (for more details of microsatellites, see [44]). Products were separated on a MegaBACE 1000 (GE Healthcare) instrument and analysed with the program Fragment Profiler (GE Healthcare).

### Precision of single cell sorting

To assess the precision of single cell sorting with the previously defined gate P1 (Fig. 1A), we mixed five strains (coming from our samples, with internal nos. 08.068, 08.075, 08.076, 08.091 and 08.161) at approximately equal numbers immediately before single cell sorting. For this purpose, cell numbers were determined microscopically using a Neubauer improved haemocytometer chamber. Cells were sorted into 96-well PCR plates (twin.tec PCR plate 96, skirted; Eppendorf, Hamburg, Germany) containing 50 µl medium. After sorting, plates were immediately closed with sterile PCR strips. Extreme care was taken to exclude any external contamination and plates were only handled on a sterile bench. Plates were then kept in an incubator at 27°C with 3% CO_2_ for two weeks. Cells were collected by centrifuging the plates at 1,000 rpm for 10 min and by removing the supernatant by shaking off the liquid. To wash the cells, 50 µl of PBS buffer was added to each well, the plate again centrifuged at 1,000 rpm and the supernatant removed. Plates were then stored at −20°C until further use. Extraction of genomic DNA was directly done in the PCR plates using DirectPCR Lysis Reagent (Cell) (Viagen Biotech; Los Angeles, USA) with Proteinase K (20 mg/ml) added. Preliminary tests showed that a 5–10 times diluted Lysis Buffer produced best results. 50 µl of this diluted lysis buffer (mix for 1 plate: 0.5 ml DirectPCR Lysis Reagent, Cell, 4.5 ml dH_2_0, 0.05 ml Proteinase K) was added to each well. Plates were then incubated at 55°C for 45 min, followed by 40 min at 85°C to inhibit Proteinase K activity. 5 µl of this DNA (total of 20 µl PCR volume) was directly used for multiplex PCR (Msat 1 and Msat 2; PCR conditions as described above).

## Results

### Initial evaluation of isolation and cultivation protocols

Several methods were tested to isolate the parasite *C. bombi* from the host gut (Table 1). All of these initial methods were tested using seven different isolates of *C. bombi* from bees collected in long-term field sites in Northern Switzerland. A major goal of the isolation protocol was to prevent contamination of the isolated cultures with bacteria and yeast, which typically would quickly outgrow *C. bombi*. The addition of antibiotics is a widely used method to achieve this goal. For a detailed description of all relevant media, see Tables in Information S1. Here, we used the ‘Mäser mix’ as an addition to cultivation media (see Information S1); usually, an addition of 2% Mäser mix to medium was sufficient. However, the antibiotics also impeded the growth of *C. bombi*.

**Table 1:** Tested classical protocols for the isolation of *C. bombi* from the host (after [38]).

Method	Procedure	Finding
Standard	Sample in medium, with addition of 2% Mäser mix. Incubation at 27°C, 3% CO_2_ for 24 h. Extraction of supernatant from 15 ml falcon tubes with 2–3 ml of medium.	*C. bombi* is enriched, but contaminated with yeast.
Density-Gradient	Stepwise gradient of 30%, 50%, 70% percoll in tubes containing medium. Extraction after centrifugation with respective speeds.	In all cases, no distinct layer of *C. bombi* could be extracted.
Serum-Gradient	In medium, with addition of 25% serum albumin, and a 10% layer on top. After centrifugation, tube cultured for 24 h (27°C, 3% CO_2_).	*C. bombi* enriched at boundary layer between serum gradients. Extractions from this layer free of yeast but difficult to perform.

To extract the parasites, host bees were freeze-killed and then washed in 70% ethanol for 3 min to clear possible external contamination by bacteria or other spores. Afterwards, the bees were dried, the gut extracted onto a sterile slide and parasites subsequently isolated in various ways. Table 1 summarizes the findings of these attempts. An old standard method, for example, makes use of the fact that flagellates (such as *C. bombi*) tend to move up to the surface when hosted in a medium-filled tube. With the density-gradient methods, parasite cells should sediment in the layer that corresponds to their own density (isopycnic sedimentation). Gradients can be generated by adding to medium either different concentrations of Percoll or serum albumin. Neither method was ever fully satisfactory, even though the serum albumin gradient resulted in extractions free of yeast (Table 1). The study of Bouquet [38] presents a method to clone *C. bombi* from these cultures, where the cell cultures were first diluted (approx. 5×10^−4^ to 5×10^−5^ cells/ml), and from this diluted suspension, cells were transferred into single wells of a 96-well microtiter plate with the tip of a sterilized, gold-plated paper clip. In the study, wells were visually inspected and only those containing a single cell were used. In the process, also modified microtiter plates were tested: plates laminated with collagen, with artificially roughened floors, or plates from different manufacturers. With these modifications the attachment of cells to the walls of the single wells was improved. In fact, cultures where cells attached, on average, grew faster than cultures with freely swimming cells. However, the effect was considered to be too unimportant for these elaborate modifications to be useful. Our new method described below was found to be superior to these “classical” protocols in terms of time, efforts, as well as being competitive in price.

### Improvement of the cultivation medium

Cultivating trypanosomatids has succeeded for a number of cases. However, due to the varied nature of this group, it is *a priori* unknown whether any such method can be adopted for another species. This proved also to be the case for *C. bombi*. We started with the most likely candidate, Mattei medium (Table 1 in Information S1); this medium was successfully used in earlier studies [37]. From this, we developed a number of new media (Table 2) before settling to the final medium whose preparation is described in Table 3. The composition of other media can be found in the Information S1.

**Table 2:** Synopsis of tests with various media used for growing *C. bombi* in culture (see Information S1) for details on the composition of media), after [38].

Medium	Modifications, additions^1^^, ^ 2	Finding
Established media	- Grace's medium	- None of these media did lead to satisfactory growth of *C. bombi*.
	- SM medium	
	- SDM-79-medium	
Mattei medium	- Standard medium	Cultivation possible with some success [37].
	- Pollen (autoclaved): 10 g/l, 30 g/l; 10% FBS added.	- Pollen known to increase parasitaemia in living hosts. No improvement in growth compared to standard medium observed. Parasites become deformed and perish.
	- Honey: 2 g/l, 6 g/l; 10% FBS added	- No improvement in growth compared to standard medium observed.
	- Bicarbonate buffer (pH 7.6): NaHCO_3_ 2.2 g/l, plus Na_2_HPO_4_.H_2_O: 5 g/l to keep osmolarity. This buffer additionally modified by adding L-arginine, or yeast extract.	- No improvement in growth compared to standard medium observed; neither for the bicarbonate-enriched medium, nor for the modifications thereof.
	- FBS: 10%, 15%, 25%	- 10% found to promote growth.
Standard medium	- Mattei-medium, modified by adding 10% FBS, and 2 µg/l haemin	- Used as the reference medium for the following modifications
	- addition of citric cycle intermediates (fumaric acid, citric acid, pyruvic acid, α-ketoglutarate:10 mM each).	- Only minimal effects on growth.
	- addition of sugars (D− fructose, maltose, D+ mannose: 10 mM each).	- Faster growth. Maximum effect with 10 and 20 mM fructose (40 mM is inhibiting).
Modified standard medium (10 mM fructose added)	- “Mix 2”, several compounds added.	- Mix 2 promoted growth. The added compounds were therefore tested separately.
	- 10 µM folic acid added	- Same positive effect as Mix2. Therefore adopted as improved medium.
Standard-FF	Standard medium, modified by adding 10 mM fructose, and 10 µM folic acid.	- Improved growth compared to Standard medium alone.
	- BME-Vitamin mix added.	- Marginal improvement in growth compared to Standard-FF.
	- PABA/Biotin-stock solution added.	- No improvement in growth compared to Standard-FF.
	- Vitamin B added	- Slight improvement in growth compared to Standard-FF.
	- Haemin added: 2, 4, 8, 16, and 32 µg/l	- Addition of 2 µg/l sufficient to promote growth compared to Standard-FF.
Haemin-enriched Standard-FF	Standard-FF with 2 µg/l haemin	
	- addition of amino acids: taurin (10 mM); L-ornithine-monhydrochloride (2.5 mM); mix of isoleucine-valine- L-ornithine-monhydrochloride (1.5 mM, 1.7 mM, 1.2 mM); L-proline (2.5 mM).	- No effects, except for L-proline.

1 FBS: Fetal Bovine Serum (Biological Industries, K Beth Haemek, Israel). FBS could not be replaced by horse serum, or calf serum, as cells agglutinated and perished in these alternatives.

2 Composition of media can be found in the following references.

- Grace (Gibco 11605) [68], developed for insect cell cultures.

- SDM-79 [69], used for cultivation of pro-cyclic forms of *Trypanosoma brucei*.

*-* SM [70], used for cultivation of trypanosomatids.

**Table 3:** Preparation of optimized culture medium for *C. bombi*.

FP Stock solution(1)	
NaCl	2.8 g
KCL	0.4 g
NaH_2_PO_4_×H_2_O	10.0 g
Tryptose Broth (Difco™)	10.0 g
Liver Infusion Broth (Difco™)	2.0 g
H_2_O bidest: fill to total volume of 1000 ml; set to pH of 5.8	
10× FB stock solution(2)	
D(-)Fructose	1.8 g
L-Proline	0.289 g
Vitamine B_1_ hydrochloride	0.0067 g
Folic acid	0.0044 g
FP stock solution to fill to final volume of 100 ml	
Heat inactivated fetal bovine serum (hiFBS)(3)	
Haemin stock (2 mg/ml)(4)	
Haemin-chloride	100.0 mg
NaOH (1 M)	1.25 ml
H_2_O bidest	23.75 ml
Dissolve by heating to boiling point, cool down and add H_2_O to final volume of 50 ml	
Full medium	
FP Stock solution	36.0 ml
10× FB stock solution,	4.0 ml
hiFBS	4.5 ml
Haemin	44.5 µl

(1) autoclave and store at 4°C.

(2) aliquot to 5.0 ml and store at −20°C.

(3) aliquot to 5.0 ml and store at −20°C.

(4) aliquot to 50 µl.

Table 2 gives an overview over the various attempts and their respective success. All modifications were chosen based on plausible knowledge for trypanosomes. For example, insect guts are often reported to have a pH of 7.5–8.0 [45]–[47] whereas pure Mattei medium has a pH 7.2. For this reason bicarbonate was added to change the acidity to pH 7.6 (see Table in Information S1). Similar reasoning applied to the other cases of Table 2, too. Growth of *C. bombi* was estimated from the slope of log-transformed growth curve (in cells/ml) during the log-phase (exponential growth); also saturation density was measured as the cell number at which the culture clearly showed reduced growth and reached a near-stationary phase. For increased precision, cell numbers were counted by visual inspection, using appropriately diluted probes as necessary. In routine use for the laboratory, this is normally replaced by measurement of optical densities, a method also described by other laboratories [39]. We also checked for the presence of bacterial endosymbionts *of C. bombi*, which are known to be present in other species of this group [48]–[51]. Their inadvertent removal by antibiotics used in the cultivation media would possibly harm the cell lines. Visual checks by staining the probes (Giemsa) did, however, not reveal any such endosymbionts; this has meanwhile also been confirmed by genetic typing (unpublished data).

The evaluation process converged to an improved medium (“FP-FB medium”; see Information S1) whose preparation is described in Table 3. Adding extra compounds to the starting Mattei medium seems justified by the biology of most trypanosomes. For example, many species appear to be unable to synthesize certain chemical groups and so need haemin, haematin, or haemoglobin in the medium. *Leishmania* (closely related to *C. bombi* [52]) and *C. oncopelti*, for example, both need heme exogenously - a well-known metabolic deficiency of trypansomatid protozoans [53]. Furthermore, the tests suggested that the appropriate temperature, pH, and CO_2_-atmosphere are all important for successful cultivation of *C. bombi*, especially at the start of the cultivation process. After evaluating different concentrations, providing an atmosphere with 3% CO_2_ proved to be the most efficient condition. Similarly, it was found that a low pH of 5.8 had the best effect, and that a temperature of 27°C (out of the range of 21°C to 37°C) was optimal.

The growth curves obtained in these trials [38] were used to estimate generation time, *T*, of *C. bombi* according to eq.1:
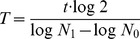
(1)where *N*_0_ is the cell number at the beginning of the log-phase, *N*_1_ the cell number at the end, and *t* the time interval between the beginning and end of the log-phase. Each curve each was obtained by averaging the growth of all successful clones in the 96-well microtiter plates (cloning success was around 70%; [38]). From those, a generation time in the range of *T* = 10–16 h was calculated.

### New method for isolation

Some of the methods described above were successful to isolate single *C. bombi* cells into microtiter plates, and for cultivation largely free of contamination. However, either the addition of antibiotics with unknown effects on *C. bombi* variants, or time-consuming technical procedures were necessary. These disadvantages could be circumvented by the method described here. We developed a method based on fluorescence-activated cell sorting (FACS). Most remarkably, in the case of *C. bombi* no fluorescent markers are needed; sorting by FACS-instruments based on cell size and surface structures alone is possible.

In particular, we found that direct sorting of single cells from natural, complex samples (i.e. host faeces) using gate P1 (Fig. 1) successfully produced clones and cultures of *C. bombi* from all of the 31 infected queens of *B. terrestris*, tested for this new method. We obtained, on average, around 40, but up to 80, clones per plate with 96 wells [54]. To demonstrate the applicability of this method, we investigated the diversity of the clones used for this study. We randomly selected one clone from each of the 31 queens, and genotyped these at five polymorphic microsatellite loci (Table 4). The clonal diversity measured by multiplex microsatellite PCR was shown to be very high. In all, 28 out of the 31 clones had a unique multi-locus genotype. Only clones nos. 08.068, 08.073 and 08.134, which had all been from queens collected at site Neunforn, had the same multi-locus genotype (Table 4).

**Table 4:** Summary of clones derived from 31 infected queens.

Clone no.	Site	Locus				
		*Cri4*	*Cri16*	*Cri2F10*	*Cri4G9*	*Cri1B6*
08.021	N	125/131	119/119	117/117	138/154	141/153
08.031	N	125/133	116/119	115/117	138/158	147/163
08.037	N	129/129	116/119	117/117	138/144	141/163
08.063	N	125/129	119/122	117/119	138/144	135/151
08.068	N	125/131	119/122	115/117	138/154	141/147
08.072	N	129/131	119/119	117/117	154/164	135/157
08.073	N	125/131	119/122	115/117	138/154	141/147
08.075	N	129/133	116/119	117/125	150/154	137/141
08.076	N	129/131	116/122	117/117	138/138	149/157
08.091	N	131/133	116/122	117/125	154/158	149/159
08.129	N	133/133	119/119	115/125	138/152	149/151
08.134	N	125/131	119/122	115/117	138/154	141/147
08.152	N	129/131	119/119	117/117	138/164	141/161
08.157	N	129/131	116/119	117/117	138/150	141/155
08.161	N	133/133	119/119	117/119	144/150	135/151
08.169	N	125/133	116/119	114/117	152/164	151/151
08.192	N	129/129	119/119	117/117	154/166	135/135
08.196	N	129/131	119/119	114/117	138/144	147/151
08.226	A	125/129	122/122	117/125	138/144	135/151
08.228	A	129/133	116/119	117/117	150/156	141/149
08.239	A	125/129	116/119	117/117	154/166	135/155
08.246	A	131/133	119/119	115/125	154/156	145/151
08.261	A	129/131	119/119	115/117	138/164	141/163
08.281	A	127/133	119/119	115/117	150/156	135/135
08.285	A	131/133	116/119	115/117	138/152	135/139
08.286	A	125/133	116/116	117/117	152/154	137/151
08.288	A	125/125	119/119	115/117	154/164	141/151
08.293	A	129/131	119/119	117/119	138/156	149/163
08.320	A	125/133	119/119	117/117	138/152	135/151
08.327	A	129/131	119/119	117/117	150/164	135/153
08.332	A	129/131	119/119	117/125	150/158	135/159

One clone per queen was analysed here to show the diversity of genotypes. Entries are alleles (fragment length in bp) at five loci. Note that *C. bombi* is diploid. Sites are Neunforn (N) and Aesch (A).

### Precision of single-cell sorting

To test for the accuracy of cell sorting, a mixture of five strains (internal code nos. 08.068, 08.075, 08.076, 08.091, 08.161) at equal proportions was processed and sorted in eight replicates (i.e. eight plates) into 96-well plates. From these plates, we were able to amplify the microsatellites and genotype the material of 651 wells (out of 8×96 = 768 wells; 85% of probes); in the remainder of the probes not all loci amplified and the results from the respective wells were thus discarded. The tested assumption is that all wells contained a single genotype (clone) only. The respective multi-locus genotyping showed that the precision of single cell sorting was indeed very high: 647 of the 651 wells contained pure clones, which could be assigned to one of the five original clones used in the experiment. Only four wells (0.62% of all probes) contained a mixture of two original clones, suggesting an overall technical error rate of <1% (Table 5).

**Table 5:** Quality control for the sorting method.

Plate^1^	Clone no.^2^					Errors^3^		Total
	08.068	08.075	08.076	08.091	08.161	double	empty	
plate 1	8	18	16	25	16	4	9	
96								
plate 2	4	12	19	19	19	0	23	96
plate 3	6	22	17	23	17	0	11	96
plate 4	6	15	21	19	22	0	13	96
plate 5	4	10	21	24	24	0	13	96
plate 6	4	19	19	23	13	0	18	96
plate 7	7	19	23	25	9	0	13	96
plate 8	4	25	20	12	18	0	17	96
Total	43	140	156	170	138	4	117	768
Mean	5.37	17.50	19.50	21.25	17.25			
S.D.	1.60	4.98	2.27	4.43	4.77			
C.V.^4^	0.297	0.285	0.116	0.208	0.277			

A standard mixture of five clones was sorted with our method and genotyped. A total of 651 wells could be genotyped.

1 Each plate is a replicate for the same mixture.

2 Clones: for genotypes, see Tab. 2.

3 Errors occur when two clones are found in a single well (double); wells without cells are not errors of the sorting process in this sense but remain empty, and thus are unambiguous.

4 Coefficient of variation.

### Long-term storage

All of the 31 parasite genotypes (Table 4) were processed to generate cryo-stabilates for long-term storage (see Methods for details) in spring 2008. We retrieved these samples after four years in April and May 2012. The samples were processes and re-cultured as described in the Methods. We found that all samples could indeed be revived and, after growing them in medium for one to two weeks, did produce healthy and viable cell cultures. Furthermore, all of the samples were used to infect workers of *B. terrestris* for a range of experiments. In all cases, the 7infection was successful. Previous studies had already suggested that cryo-stabilization allows to keep clones for prolonged periods of time while remaining infective [36]–[38]. This has now been confirmed for at least a period of four years.

## Discussion

The natural environment of *C. bombi* - the gut of bumblebees - is also home to many bacteria and fungi [55], . This makes specific cultivation of *C. bombi* and other comparable parasites of insects notoriously difficult [48], [57] because the loss of cultures due to contamination is usually very high. Establishing cultures from environmental samples using flow cytometry has been previously described for several organisms (e.g. [58] including particularly fragile and difficult organisms such as dinoflagellates [59]. Flow cytometry is known to allow the sorting of single or multiple cells based on fluorescent labelling [60]. However, here we show that we can sort *C. bombi* and thereby separate the parasite from a complex background of other particles and organisms, as is typical for natural samples, based simply on specificity in size and cell surface structure (see gates in Fig. 1). Furthermore, single cells can be directly sorted into growth medium in a highly efficient and accurate way (Table 3) such that large number of cultures can subsequently be established from these cells. The specific sorting process thus eliminates the problem of contamination with bacteria and fungi from natural samples, and therefore allows for establishment of cultures without the addition of any antibiotics that typically impede growth of *C. bombi*. Our study hence demonstrates that establishing cultures from faeces using flow cytometric single cell sorting from bumblebee faeces can successfully be applied to obtain clean and healthy cultures of *C. bombi*. Furthermore, we were able to obtain viable clones from all infected hosts (Table 3). All of the wells of the precision experiment erroneously containing two clones were sorted with the first plate, perhaps being a sign of machine warm-up. Empty wells could either have resulted from possible post-sorting cell death or from an over-conservancy of the technique.

Looking at the clones in our samples, we conclude that the majority of hosts that we obtained faeces from (28 out of 31) turned out to be infected by distinct *C. bombi* multi-locus genotypes (which is a clone by definition) (Table 2). Only three hosts carried the same-clone genotype. This finding suggests that almost every spring queen carries her individual, specific genotype. This is not surprising considering that it has recently been shown that in field populations of bumblebees a particular multi-locus genotype of an infection with *C. bombi* is hardly found more than once [30]. In that study we had also found approximately half of the *C. bombi* infections in the field to be of mixed genotypes.

As detailed in the results, it is worth emphasizing that the suggested new method is the result of a lengthy process starting with the use of Mattei-Medium [37] and subsequent modifications. Several methods were tested during this process and found to be inadequate; these are described in detail in [38]. Inadequate methods were, for example, the direct isolation of *C. bombi* from faeces with subsequent growth in medium enriched with antibiotics to eliminate contaminating micro-organisms; this method is still in use [39]. None of these procedures improved the results to the extent that it defined a more efficient method than described here [38]. In fact, FACS-assisted sorting in combination with FP-FB-medium as used here, and appropriate physical-chemical conditions (27°C, pH 5.8, 3% CO_2_) proved to have the best success across a range of tested *C. bombi* isolates. Note that this best temperature is within the range of temperatures typical for a nest (28–32°C) and for a flying bee [61], [62]. Acidic conditions have also been identified as optimal when *C. mellificae* was first described [63].

Recently, Popp and Lattorff [39] described an isolation/cultivation method with medium that generated comparable growth curves for *C. bombi* as reported earlier for experimental infections [38], [64]. Their cultivation medium (BHI-brain-heart infusion medium developed for *C. fasciculata* [65]) differs from ours reported here (Table 3), which we have now used successfully in the laboratory since 2004. In addition, [39]'s medium had to be enriched with a complex mixture of antibiotics to eliminate contamination. According to observations in our own laboratory, such antibiotics are known to massively impede growth and eventually kill clonal lines of *C. bombi* at the relevant dosages needed to keep natural samples free of contaminants. In contrast to our method, which readily allows the long-term propagation of clonal lines, [39] plated the infections on agar plates, which then requires picking single growth units for further propagation. Unfortunately, in their study, no verification of the accuracy of the process, the stability of propagated clones, or the success rate of the procedure was reported.

Long-term cryopreservation has also been demonstrated for several other trypanosomes, such as *T. brucei* [66]. We here show that *C. bombi* clones remain infective after prolonged storage at −80°C [34], [36], [42], and that this storage can be extended for a period of at least 4 years. Last but not least, we have recently shown that clones remain genetically stable during long-term live cultivation (over 10 months [35], which is also known to be feasible for *Trypanosoma evansi* [67], albeit with occasional loss of alleles, but continued recognizable identity based on multi-locus genotypes.

## Supporting Information

Information S1 **Media for the cultivation.** Table 1: Mattei medium (after Mattei et al. 1977, and Camargo 1964). Table 2: List of compounds added to high pH carbonate medium. Table 3: “Standard medium” (see text). Table 4: List of compounds added to “Standard medium”. Table 5: List of compounds added to “Standard -FF medium”. Table 6: Standard “Mix-2” medium (see text). Table 7: Mäser mix of antibiotics (after Mäser et al. 2002). Table 8: Medium for the cultivation of *C. bombi* (FP-FB medium).
(DOCX)
