# Supplementary material for: Probing Mixed-Genotype Infections I: Extraction and Cloning of Infections from Hosts of the Trypanosomatid Crithidia bombi
Source: PLoS One. 2012 Nov 14;7(11):e49046. doi: 10.1371/journal.pone.0049046 (PMC3498296; doi:10.1371/journal.pone.0049046)
Supplement: Information S1 — Media for the cultivation. Table 1: Mattei medium (after Mattei et al. 1977, and Camargo 1964). Table 2: List of compounds added to high pH carbonate medium. Table 3: “Standard medium” (see text). Table 4: List of compounds added to “Standard medium”. Table 5: List of compounds added to “Standard -FF medium”. Table 6: Standard “Mix-2” medium (see text). Table 7: Mäser mix of antibiotics (after Mäser et al. 2002). Table 8: Medium for the cultivation of C. bombi (FP-FB medium). (DOCX) [file pone.0049046.s001.docx]

SUPPORTING INFORMATION S1

**Media for the cultivation**

from: Bouquet, C. 2003. In vitro cultivation and cloning of *Crithidia bombi*. University of Basel, Diploma Thesis, Swiss Tropical Institute.

**Table 1**

| Compound | Amount |
| --- | --- |
| NaCl | 4.0 g |
| KCl | 0.4 g |
| Na_2_HPO_4_ H_2_O | 10.0 g |
| Glucose | 2.0 g |
| Tryptose (Difco) | 10.0 g |
| Liver infusion broth (Difco) | 2.0 g |
| H_2_O bidest. | for 1'000 ml |
| pH | 7.2 |

**Table 2**

| Compound | Amount |
| --- | --- |
| L-arginine HCl | 1 mM, 2mM, 3mM |
| Yeast extract  (Becton Dickinson 211929) | Substitution of 5 g/l tryptose with 5 g/l yeast extract (only 5 g/l tryptose remains) |

**Table 3**

| Compound | Amount |
| --- | --- |
| NaCl | 43.0 g |
| KCl | 0.4 g |
| Na_2_HPO_4_ H_2_O | 10.0 g |
| Tryptose (Difco) | 10.0 g |
| Liver infusion broth (Difco) | 2.0 g |

**Table 4**

| Compound | Amount |
| --- | --- |
| Folic acid (Fluka 4762) | 10 µM |
| Thiamine dichloride (Merck 8181) | 20 µM |
| BME-Vitaminmix 100x (Sigma B 6891) | 5 ml/l |
| NCTC-Vitaminmix for amoeba | 10 ml/l |

**Table 5**

| Compound | Amount |
| --- | --- |
| **Citric cycle intermediates:** |  |
| Na_3_-citrate-3 H_2_O | 10 mM |
| α-ketoglutarate (Merck 5194) | 10 mM |
| Na-pyruvate (Merck 6619) | 10 mM |
| Di-Na^+^ fumarate (Fluka 47970) | 10 mM |
| **Sugars:** |  |
| D(+) glucose (Fluka 49140) | 11 mM |
| D(-) fructose (Merck 5323) | 10, 20, 40 mM |
| D(+) mannose (Sigma M-6020) | 10 mM |
| Maltose - H_2_O (merck Microbio 5910) | 10 mM |
| **Amino acids:** |  |
| L-Proline (Fluka 81709) | 2.5 mM |
| DL-isoleucine (Fluka 58885) | 1.5 mM |
| L-valine (Fluka 94620) | 1.7 mM |
| L-ornithine-monohydrochloride (Merck 6906) | 1.2 mM |
| Di-Na+ taurine | 10 mM |

**Table 6**

| Compound | Amount |
| --- | --- |
| D(-) fructose | 10 mM |
| L-valine | 1.7 mM |
| L-ornithine-monohydrochloride | 1.2 mM |
| DL-isoleucine | 1.5 mM |
| Taurine | 10 mM |
| Folic acid | 10 µM |
| Thiamine dichloride | 20 µM |
| BME-Vitamin mix | 1 % |

**Table 7**

| Compound | Preparation | Amount |
| --- | --- | --- |
| Penicillin (Sigma) |  | 6 mg/ml |
| Kanamycinn (Fluka 60615) |  | 10 mg/ml |
| Fluorcytosin (Fluka 46850) | dissolve in H_2_O bidest. | 5 mg/ml |
| Chloramphenicol (Fluka 23275) | dissolve in 70% ethanol | 1 mg/ml |

**Table 8**

| Compound | Amount |
| --- | --- |
| NaCl | 2.8 g |
| KCl | 0.4 g |
| Na_2_HPO_4_ H_2_0 | 10 g |
| D(-) fructose | 1.8 g (10 mM) |
| L-proline | 289 mg (2.5 mM) |
| Tryptose (Difco) | 10 g |
| Liver infusion broth (Difco) | 2 g |
| Thiamine dichloride | 6.7 mg (20 µM) |
| Folic acid | 4.4 mg (10 µM) |
| pH adjust with 4N HCl | 7.0 / 5.8 see text |
| H2O bidest., add up to | 1'000ml |

Store at -20˚C. For full medium, add FBS (10 %), and haemin (2 µg/ml).

**References**

Camargo, E. P. 1964. Growth and differentiation in *Trypanosoma cruzi*. Revista do Instituto de Medicina Tropical de São Paolo 6: 93-100.

Mäser, P., Y. Grether-Bühler, R. Kaminsky, and R. Brun. 2002. An anti-contamination cocktail for the in vitro isolation and cultivation of parasitic protozoa. Parasitology Research 88: 172-174.

Mattei, D. M., S. Goldenberg, C. Morel, H. O. Azevedo, and I. Roitman. 1977. Biochemical strain characterization of Trypanosoma cruzi by restriction endonuclease cleavage of kinetoplast-DNA. FEBS Letters 74: 264-268.
